# A cross-sectional survey on VEXAS syndrome: insights from a global expert panel

**DOI:** 10.1007/s10067-025-07617-3

**Published:** 2025-08-09

**Authors:** Syed B. Ali, Carmelo Gurnari

**Affiliations:** 1 Department of Clinical Immunology and Allergy, Flinders Medical Centre, Bedford Park, South Australia Australia; 2 School of Medicine and Public Health, Flinders University, Bedford Park, South Australia Australia; 3 School of Medicine and Biomedical Sciences, University of Adelaide, Adelaide, South Australia Australia; 4 Department of Translational Hematology and Oncology Research, Taussig Cancer Institute, Cleveland Clinic, Cleveland, OH USA; 5 Department of Biomedicine and Prevention, University of Rome Tor Vergata, Rome, Italy

**Keywords:** Management, Physician practices, Questionnaires, Surveys, VEXAS

## Abstract

**Background:**

Vacuolization, E1 enzyme, X-linked, autoinflammatory, somatic (VEXAS) syndrome is recently described, for which the diagnosis and management lack official guidelines.

**Aims:**

To assess the diagnostic capabilities and disease management of VEXAS syndrome among physicians in the global context.

**Methods:**

An electronic survey was sent to clinicians with expertise in VEXAS syndrome between January and February 2025 to gather real-life data on the management of VEXAS.

**Results:**

Seventy-four clinicians completed the survey from Europe (*n* = 51, 68.9%), North America (*n* = 9, 12.2%), Australasia (*n* = 6, 8.1%), Asia (*n* = 6, 8.1%), Africa (*n* = 1, 1.4%), and South America (*n* = 1, 1.4%), mostly being hematologists (*n* = 24, 32.4%) and rheumatologists (*n* = 24, 32.4%). Majority of the clinicians were managing between 1 and 4 (*n* = 40, 54.1%) and 5 and 9 patients (*n* = 17, 23%) with VEXAS syndrome, with regular clinic review, typically under 7-weekly intervals (*n* = 44, 59.5%).

*UBA1* mutation testing was available for 76% of physicians and next-generation sequencing (NGS) of the entire gene was most common (*n* = 24, 32.9%) with a turnaround time within 12 weeks. C-reactive protein (CRP) was selected by over half of the clinicians (*n* = 35, 55.4%) as a marker of disease relapse. Treatment with corticosteroids at 1 mg/kg (*n* = 48, 64.9%) was the most common initial dosing and upfront systemic immunomodulatory treatment was added by more than half of clinicians (*n* = 39, 52.7%). The most frequent treatments of choice (*n* = 66) were Janus kinase inhibitors (JAKi) and IL-6 targeted monoclonal antibodies (both *n* = 21, 31.8%). Azacitidine was mostly used in patients with concomitant myelodysplastic syndrome (MDS) (69.9%). Only 29.6% indicated that allogeneic hematopoietic stem cell transplant (allo-HSCT) had been successfully completed in their department.

**Conclusions:**

This is the first clinician survey on VEXAS syndrome, encompassing a global representation of multiple specialties on current disease management, highlighting several unmet needs (longitudinal follow-up, lack of on-label drugs, financial toxicity) actionable for future research.

**Table:** 

**Keypoints** • *Globally, VEXAS syndrome is being increasingly recognized, and this survey demonstrates real-life physician practices.* • *The survey identified both hematologists and rheumatologists as the main care providers, amongst other specialties, identifying the need for a multidisciplinary approach in managing patients with VEXAS syndrome.* • *Diagnostic UBA1 testing was available for more than 75% clinicians with turnaround time < 12 weeks and C-reactive protein was selected as a useful marker of disease relapse.* • *Treatment modalities were heterogeneous, identifying the need for consensus guidelines.*

**Supplementary Information:**

The online version contains supplementary material available at 10.1007/s10067-025-07617-3.

## Introduction

Vacuolization, E1 enzyme, X-linked, autoinflammatory, somatic (VEXAS) syndrome is a recently described disease entity arising due to a somatic mutation in the ubiquitin-activating enzyme (*UBA1*) on the X-chromosome [1]. The *UBA1* enzyme is involved in many cellular functions such as protein homeostasis and cell signaling. Dysregulation of the ubiquitination process results in uncontrolled inflammation [2, 3].

Given the recent description and involvement of *UBA1* in diverse cellular pathways, diagnosis can be especially challenging because patients can present with heterogenous manifestations. Additionally, VEXAS syndrome can coexist with myelodysplastic syndrome (MDS), for which targeted therapy may have already been initiated. Currently, there are no specific diagnostic criteria except for the presence of the defining *UBA1* somatic mutation. Consequently, factors such as availability of *UBA1* mutation testing and the absence of one specific managing specialty add further complexity to the diagnosis and management of VEXAS syndrome.

Further awareness of the disease is clearly needed, as patient comorbidities can obscure or complicate the diagnosis of VEXAS syndrome. The delay in diagnosis has been reported to be up to 6 years with the risk of accumulating morbidity in patients during this time period [4].

In the absence of large-scale prospective cohorts with longitudinal follow-up, there is a paucity of data to inform treatment algorithms and guidelines. While corticosteroids remain an integral part of therapy, prolonged treatment is often required, leading to an array of complications such as metabolic syndrome and a higher risk of opportunistic infections [5, 6]. Targeted cytokine treatments aim to diminish inflammation which include biological monoclonal antibodies and oral Janus kinase inhibitors (JAKi) [7]. Many, if not all, are not available routinely as no “on-label” indication exists thus far, resulting in the additional complexities of pursuing compassionate access schemes. Finally, allogeneic hematopoietic stem cell transplantation (allo-HSCT) has been successfully completed in some cases but access, experience, and patient factors (age and comorbidities) may preclude transplant for many patients [8].

To evaluate the experience of clinicians globally and gather information on real-life practices on a novel disease for which no guidelines exist, a survey was initiated regarding the diagnosis and management of VEXAS syndrome. The purpose of this survey was to provide a screenshot of the global VEXAS cartography in terms of diagnostic capabilities and disease management.

## Methods

As this is the first clinician survey on VEXAS syndrome, we first identified potential clinically relevant topics by searching all VEXAS literature since the first seminal description in 2020 until December 2024 (total of 412 PubMed items).

There were 42 questions created for the survey, grouped into seven sections: clinician background (two questions), clinical cohort (four questions), molecular biology/*UBA1* testing (10 questions), thrombosis and associated laboratory testing (four questions), ancillary laboratory and clinical investigations (five questions), and management of VEXAS syndrome (17 questions) (see also SupplementaryMaterial for the full survey).

Questions were a combination of either multiple choice or short answers to comprehensively cover all topics and encourage easy completion of the survey [9]. While most questions were mandatory, there were some which were optional. The initial questionnaire was pilot tested by two clinicians not involved in study design, and their feedback was used to formulate the final version. The survey was created and distributed using Google Forms. In pilot testing, the survey took 15 minutes to complete.

Purposive and convenience sampling strategies were employed. Clinicians with internationally recognized expertise in VEXAS syndrome were invited via email to complete the survey and share with other interested clinicians. To broaden the geographical coverage of the survey, aiming to reflect global perspectives, additional clinicians were identified by searching the literature on VEXAS syndrome and directly contacting corresponding authors with a link to complete the survey. All invitations included a short preamble that clearly specified the goals of the survey, its target respondents, and contact details of the investigators for further inquiries [9].

Clinician respondents were only able to begin the survey after a mandatory consent prompt. The survey was approved by the Southern Adelaide Clinical Human Research Ethics Committee (SAC HREC, reference number: 239.4).

The survey was open for completion for approximately 6 weeks (January 21 to February 28, 2025). Descriptive statistical analyses of the data were completed using Microsoft Excel (Version 16.98) and GraphPad Prism (Version 10.4.1). All metrics in the study were generally treated as categorical with frequencies and distributions expressed as percentages.

## Results

### Clinician background and experience with VEXAS syndrome

**Fig. 1:**
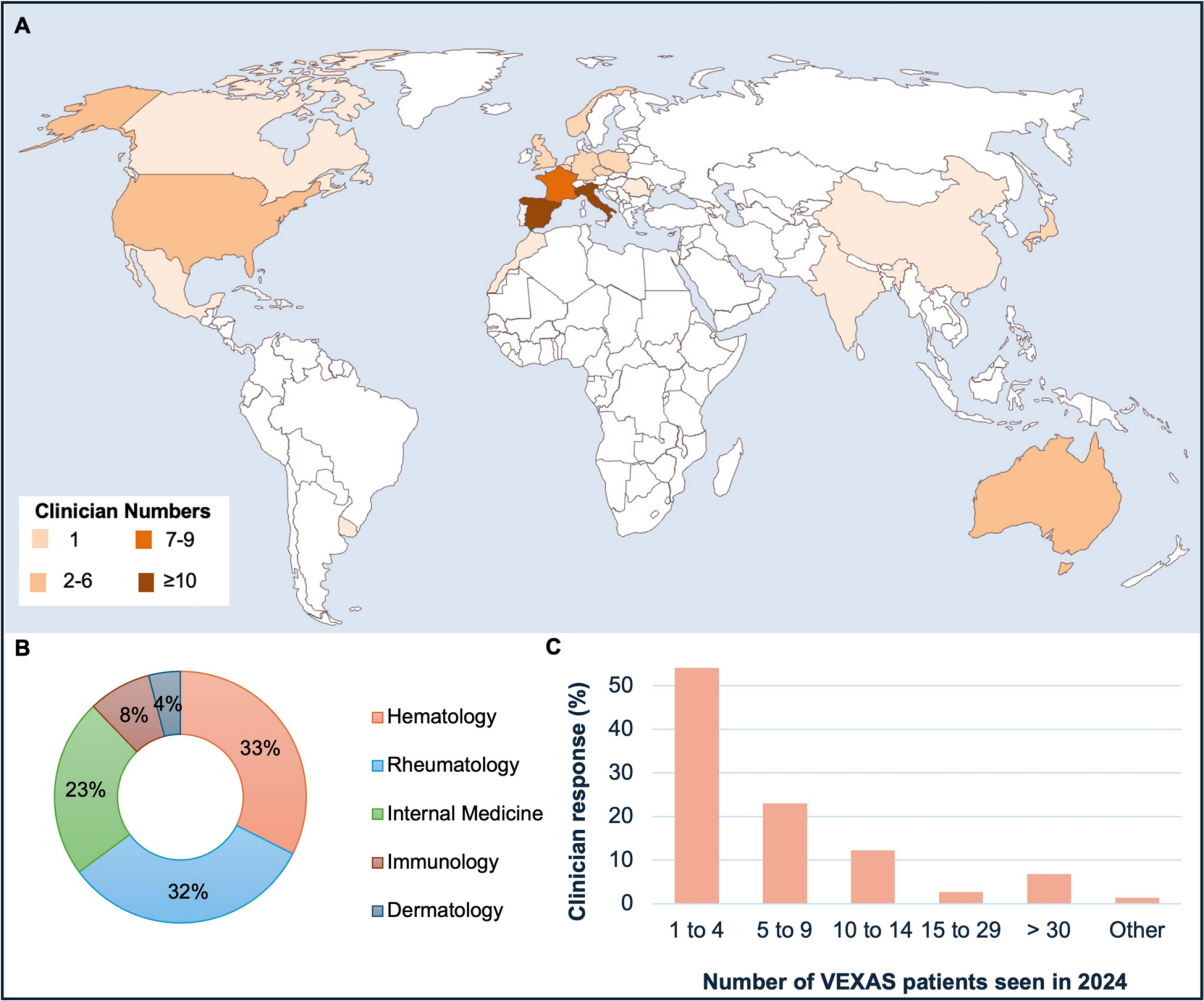
VEXAS syndrome clinician survey global cartography. **A** Geographical representation of clinicians who participated in the VEXAS survey including numbers. **B** Clinician practicing specialties. **C** Number of patients with VEXAS syndrome managed by the clinicians in 2024

A total of 74 clinicians completed the survey with geographic representation as follows: Italy (*n* = 19, 25.7%), Spain (*n* = 10, 13.5%), France (*n* = 8, 10.8%), the USA (*n* = 6, 8.1%), Australia (*n* = 6, 8.1%), Canada (*n* = 2, 2.7%), Germany (*n* = 2, 2.7%), India (*n* = 2, 2.7%), Japan (*n* = 2, 2.7%), Norway (*n* = 2, 2.7%), Poland (*n* = 2, 2.7%), Portugal (*n* = 2, 2.7%), UK (*n* = 2, 2.7%), and one clinician each from Belgium, Czech Republic, China, Israel, Morocco, Mexico, Romania, The Netherlands, and Uruguay (Fig. 1A and Table S1).

The clinicians were physicians in the following specialties: hematology (*n* = 24, 32.4%), rheumatology (*n* = 24, 32.4%), internal medicine (*n* = 16, 21.6%), immunology (*n* = 6, 8.1%), and other (*n* = 4, 5.4%) (Fig. 1B).

In 2024, more than half of the clinicians managed 1–4 patients (*n* = 40, 54.1%) with VEXAS syndrome, with fewer clinicians managing 5–9 (*n* = 17, 23%), 10–14 (*n* = 9, 12.2%), 15–29 (*n* = 2, 2.7%), and > 30 (*n* = 5, 6.8%) patients (Fig. 1C). One clinician did not provide an estimated number seen. Patients with VEXAS syndrome were reviewed at the following intervals: < 4 weekly (*n* = 6, 8.1%), 4–5 weekly (*n* = 22, 29.7%), 6–7 weekly (*n* = 16, 21.6%), 8–9 weekly (*n* = 18, 24.3%), and > 10 weekly (*n* = 12, 16.2%). Notably, 58% of the respondents regularly reviewed patients with another medical specialist in a dedicated multidisciplinary clinic, with the most common combination being hematology and rheumatology (84%). Additional co-management with dermatology was reported by six clinicians.

### UBA1 testing

**Fig. 2:**
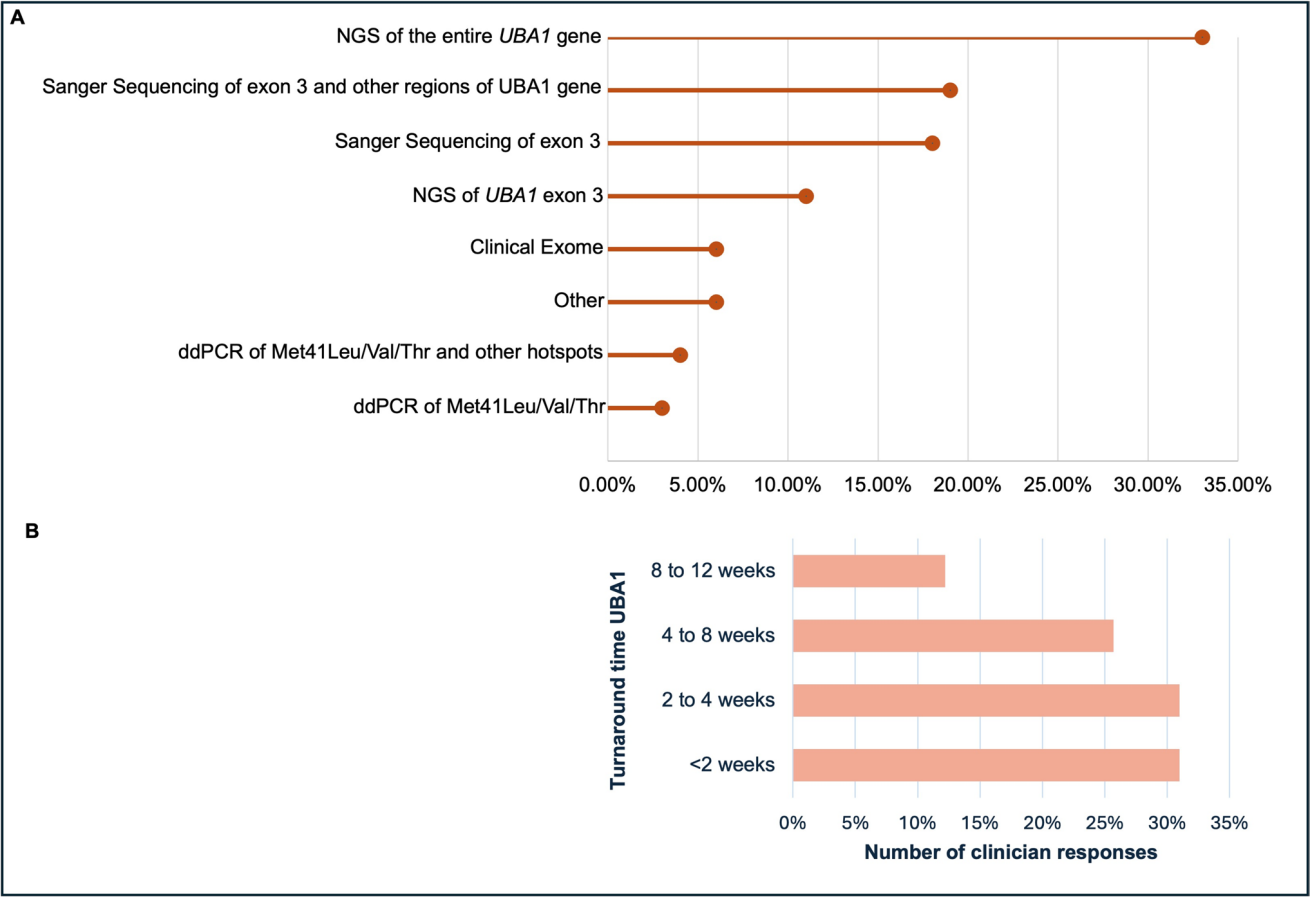
VEXAS syndrome somatic mutation investigation—*UBA1 *mutation testing and turnaround times*.* **A** Lollipop graph indicating types of molecular testing performed. **B** Turnaround times for *UBA1* mutation request by the clinician

*UBA1* diagnostic testing was performed in three quarters of the respondents’ institutions (*n* = 56, 75.7%). There was heterogeneity in *UBA1* mutation detection methods with next-generation sequencing (NGS) of the entire *UBA1* gene being the most common technique (*n* = 24, 32.9%), followed by Sanger sequencing of exon 3 and other regions of *UBA1* gene (*n* = 14, 19.2%) and specific Sanger sequencing of exon 3 (*n* = 12, 16.4%, Fig. 2A).

*UBA1* testing was more commonly performed on both peripheral blood and bone marrow (*n* = 47, 63.5%) than on peripheral blood (25, *n* = 33.8%) or bone marrow alone (2, 2.7%). Regarding turn-around times, all *UBA1* mutation results were reported within 12 weeks of request; < 2 weeks (*n* = 23, 31.1%), 2–4 weeks (*n* = 23, 31.1%), 4–8 weeks (*n* = 19, 25.7%), and 8–12 weeks (*n* = 9, 12.2%) respectively (Fig. 2B). Variant allele frequency (VAF) was offered in over half of the respondents’ institutions (*n* = 41, 55.4%). Most clinicians performed VAF testing only at diagnosis only (*n* = 22, 31.9%).

### Thrombosis and antiphospholipid antibody testing

Regarding thrombosis management, more than half of the responding clinicians used both direct oral anticoagulants (DOAC) and vitamin K antagonists (*n* = 42, 56.8%), while the remainder used either DOAC (*n* = 19, 25.7%), vitamin K antagonist alone (*n* = 5, 6.8%) or other medication, not specified (*n* = 8, 10.8%). Duration of anticoagulation in VEXAS syndrome was frequently determined according to the type of thrombosis (provoked/unprovoked) and guided by thrombophilia panels (*n* = 37, 50%). Thirty-one clinicians (41.9%) selected an indeterminate duration of anticoagulation.

Antiphospholipid antibody (APLAb) testing at VEXAS syndrome onset was requested by less than half of the clinicians (*n* = 34, 45.9%). The remainder did not request routinely (*n* = 37, 50%), had testing previously performed by others (*n* = 1, 1.4%), tested for research purposes only (*n* = 1, 1.4%), or only tested if thromboses developed (*n* = 1, 1.4%). If APLAb were found to be positive on two occasions separated by 4 months, 50% (*n* = 37) of clinicians stopped DOAC and switched to vitamin K antagonists, while 24.3% (*n* = 18) preferentially continued DOAC in the context of underlying VEXAS syndrome.

### Laboratory testing and imaging at onset and follow-up

All clinicians assessed both C-reactive protein (CRP) and full blood count at each visit (*n* = 74, 100%), with almost all respondents also regularly checking hepatic and renal function (*n* = 72, 97.3%). Glucose metabolism and erythrocyte sedimentation rate (ESR) were tested less frequently (*n* = 46, 62.2%).

Regarding the assessment of disease activity in VEXAS syndrome, CRP was the preferred biochemical marker by over half of the clinicians surveyed (*n* = 35, 55.4%), followed by full blood count (*n* = 15, 20.3%) and ESR (*n* = 10, 13.5%).

Notably, more than three quarters of the clinicians performed imaging on patients only if clinically indicated (*n* = 59, 79.7%). Further elaboration on imaging was completed by few respondents: ultrasound (*n* = 2), computer tomography (CT) *(n* = 12), and positron emission tomography (PET) scans (*n* = 5).

### Management of VEXAS

#### Corticosteroids

**Fig. 3:**
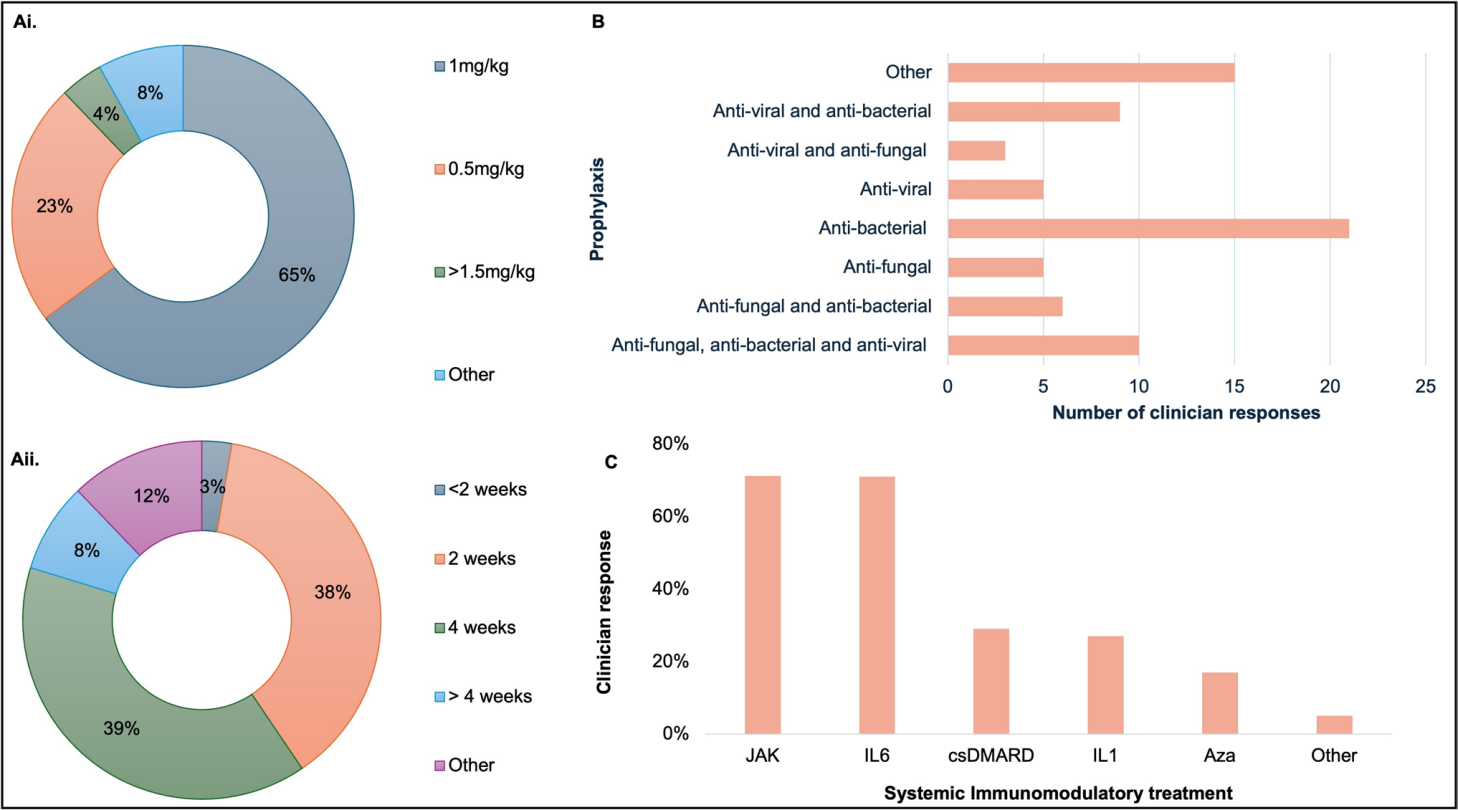
Management—VEXAS syndrome corticosteroid treatment, prophylaxis, and systemic immunomodulatory treatment. **A i** Initial daily dosing of corticosteroids and **ii** maximum time used at the highest dose. **B** Antimicrobial prophylaxis. **C** Systemic immunomodulatory treatment

Initial daily dosing of prednisolone (or equivalent) at 1 mg/kg (*n* = 48, 64.9%) was the most common treatment strategy, followed by 0.5 mg/kg (*n* = 16, 21.6%). The maximum duration of treatment with the highest dose of prednisolone (or equivalent) was generally 2 weeks (*n* = 28, 37.8%) to 4 weeks (*n* = 29, 39.2%), with a minority being treated for more than 4 weeks (*n* = 6, 8.1%) (Fig. 3A). Thirty-one (42.5%) of the respondents indicated their patients were on long-term (i.e., > 6 months) daily prednisolone (or equivalent). The overall prednisolone (or equivalent) dosing was variable: 5 mg (*n* = 6, 8.2%), 10 mg (*n* = 33, 45.2%), 15 mg (*n* = 15, 20.5%), 20 mg (*n* = 14, 19.2%), and other (*n* = 5, 6.8%). In acute disease flares, clinicians (*n* = 73) selected corticosteroids which were either oral (*n* = 31, 42.5%) or intravenous (*n* = 12, 16.4%) or both (*n* = 29, 41.1%) depending on severity of flare.

### Prophylaxis

Of the 73 clinicians who responded to the antimicrobial prophylaxis question, the most common strategy involved antibacterials alone (*n* = 21, 28.8%), followed by other (*n* = 15, 20.5%), combination antibacterial, antifungal, and antivirals (*n* = 10, 13.7%), and antivirals alone (*n* = 9, 12.3%, Fig. 3B).

### Systemic immunomodulatory treatment

Systemic immunomodulatory treatment was used upfront by more than half of the clinicians (*n* = 39, 52.7%), while the remainder introduced systemic immunomodulators if unable to wean corticosteroid monotherapy (*n* = 35, 47.3%). From 66 respondents, the most frequent treatments of choice were JAKi (*n* = 21, 31.8%) or IL-6-targeted monoclonal antibodies (*n* = 21, 31.8%), followed by conventional synthetic disease-modifying antirheumatic drugs (csDMARDS, *n* = 12, 18.2%), azacitidine (*n* = 5, 7.6%), and IL-1-targeted monoclonal antibodies (*n* = 3, 4.5%, Fig. 3C). A large proportion of clinicians trialed these systemic treatments for 3–6 months (*n* = 45, 60.8%) or 1–2 months (*n* = 24, 32.4%) before declaring inefficacy and switching to an alternate agent.

Further information for specific JAKi used was answered by 56 clinicians, for which ruxolitinib was most frequent (*n* = 28, 50%) with the 5 mg twice daily and 10 mg twice daily regimens employed by 12 and 9 clinicians, respectively. Other JAKi use included tofacitinib (*n* = 6), baricitinib (*n* = 1), upadacitinib (*n* = 2), and filgotinib (*n* = 1). Of the 67 clinician responses regarding JAKi prescription, half were obtained through a special access scheme (*n* = 34, 50.7%), followed by compassionate access (*n* = 13, 19.4%), other (*n* = 13, 19.4%), patient payment (*n* = 6, 9%), and company provision (*n* = 1, 1.5%).

Azacitidine (*n* = 73 responses) was used mostly in patients with concomitant MDS (*n* = 51, 69.9%). For those without MDS (43 responses), most received this drug through a special access scheme (*n* = 26, 60.5%), compassionate access (*n* = 8, 18.6%), patient payment (*n* = 7, 16.3%), or company provision (*n* = 2, 4.7%).

### Transplantation

Overall, 29.6% (n = 21 of 71 responding clinicians) indicated that allo-HSCT had been successfully completed at their institution for VEXAS syndrome. When asked whether they would consider allo-HSCT also in cases of VEXAS syndrome without MDS (*n* = 31 responders), 22 (*n* = 71%) responded yes.

## Discussion

To our knowledge, this is the very first clinician survey on VEXAS syndrome. Since initial discovery 5 years ago, the number of case reports and series on VEXAS syndrome has steadily risen. A recent systematic review on the clinical features of VEXAS syndrome analyzed 720 patients from 32 countries, highlighting the many varied clinical manifestations of this unique, multi-organ disease [10]. Despite the ongoing description of disease phenotype, consensus guidelines for diagnosis and treatment are lacking. This survey provides unique clinician insights into the diagnosis and management of VEXAS syndrome across varied geography, healthcare systems, and medical specialties.

A large proportion of the clinicians responding to the survey belonged to hematology, rheumatology, or internal medicine, with fewer immunology and other specialties being represented. In many institutions, both rheumatologists and immunologists are actively involved in the management of inflammatory symptoms, while hematology input is needed for concomitant MDS and consideration for allo-HSCT.

The results of this survey highlight that many clinicians do not manage large numbers of patients with VEXAS syndrome despite more widespread *UBA1* testing availability. Indeed, approximately half of the clinicians reported managing between 1 and 4 patients. Therefore, promoting global awareness of VEXAS syndrome and the broad indications for *UBA1* genetic sequencing is of key importance [11]. Delays in diagnosis of VEXAS syndrome may lead to end organ complications and accumulating morbidity, particularly if concurrent MDS is present. Our experience of VEXAS syndrome has been that of frequent clinical reviews, as patients are often refractory to treatment and can develop complications both from disease progression and also from treatments, especially given reliance on persistently high doses of corticosteroids. In the present survey, 60% of clinicians reviewed their patients within 7-weekly intervals, highlighting the importance of regular contact to assess progress, modify treatment plans, and prevent complications. Although rare, VEXAS syndrome exerts a significant burden on individuals’ life expectancy, quality of life, and work capacity, as well as impacting on the health care system due to multiple health care contacts, both inpatient as well as outpatient, distributed between multiple different medical specialties. Collaborative approaches may provide streamlined care for patients with VEXAS syndrome, although multidisciplinary clinics must balance efficacy with efficiency, and need appropriate staffing for a condition which is rare and requires subspecialist input.

In our data, *UBA1* diagnostic testing was available in > 75% of the respondents’ institutions with considerable heterogeneity in the method of detection, indicating that standardization is needed. Novel somatic variants may be missed if the entire *UBA1* gene is not tested and this might be relevant particularly in the rarer female presentations of VEXAS syndrome [12]. Furthermore, a third of the clinicians tested *UBA1* only on peripheral blood, which is consistent with the literature around the comparably more complicated procedure of bone marrow biopsy [13]. Turnaround times of *UBA1* testing were generally within 12 weeks, with just under a third being under 2 weeks, highlighting the importance of efficient and effective *UBA1* testing in confirming a diagnosis of VEXAS syndrome and initiating treatment in a timely manner. The cost of genetic testing remains a barrier for many and might explain why VAF is only being completed at diagnosis alone, despite having potential for guiding clinical management [13]. Whether VAF can be used as a marker of disease activity is yet to be determined, although there is growing evidence that reduction in VAF may indicate treatment response [14–16]. Recently, a cost-effective, mass spectrometry-based assay has been described with high accuracy, sensitivity, and specificity which may address the access and economic barriers associated with current *UBA1* testing [17].

Thrombosis as a complication of VEXAS syndrome is well recognized with a reported incidence of venous and arterial thromboses of 34.4% and 1.6%, respectively [18]. In the largest case series of patients with VEXAS syndrome (*n* = 119) reported to date, 49% had thromboses, of which venous thromboembolism comprised the majority at 41% [19]. Currently, there are no guidelines dictating duration of anticoagulation or whether having VEXAS syndrome-associated thromboses requires lifelong anticoagulation. Importantly, about 40% indicated that this duration was indeterminate given history of VEXAS syndrome. Thrombotic complications despite anticoagulation have also been reported, suggesting that VEXAS syndrome has a profoundly prothrombotic effect on multiple coagulation pathways. The pro-inflammatory drive from defects in ubiquitination, complicated by dysfunction in cellular and molecular pathways of coagulation as well as endothelial dysfunction, is likely to all contribute to VEXAS-related thrombogenesis [19, 20]. Ameliorating inflammation in VEXAS syndrome might decrease thrombosis risk, in a fashion similar to paroxysmal nocturnal hemoglobinuria (PNH), arguing against lifelong anticoagulation in patients who achieve complete control of VEXAS syndrome with treatment [21].

Survey respondents reported that frequent clinical visits for patients with VEXAS syndrome were also accompanied by regular blood test monitoring with full blood count, CRP, and hepatic and renal functions being the most requested tests. With regards to overall disease activity, CRP was the most used marker of disease activity, followed by full blood count. Fortunately, all these tests are readily available in diagnostic laboratories.

Corticosteroids remain the backbone of treatment in VEXAS syndrome, particularly for those without concurrent MDS. As observed in the present survey, a large proportion of clinicians start with high doses of prednisolone (or equivalent) at 0.5–1 mg/kg, and of these, a high proportion continue for a 4-week period. As all patients with VEXAS syndrome were reported to be on long-term steroid treatment, with 40% on doses between 15 and 20 mg, the data emphasizes the recalcitrant nature of the disease and the need for effective steroid-sparing strategies to diminish glucocorticoid toxicity [22]. Indeed, a major concern is chronic immunosuppression and infection risk with further additional immunosuppressant use. As observed, there is variability in antimicrobial prophylaxis practices which further adds a layer of complexity in harmonizing treatment strategies and avoiding drug interactions. Emerging evidence indicates that VEXAS syndrome itself may cause an underlying predisposition to infection with a large case series reporting a high rate of atypical infections even in patients on no-immunosuppressive treatment or daily glucocorticoid dosing under 10 mg prednisolone equivalent [23].

Cytokine directed therapies such as JAKi and anti-IL-6 monoclonal antibodies were most frequent non-glucocorticoid immunosuppressants used in VEXAS syndrome. A recent systematic review and meta-analysis evaluating such treatments concluded that complete responses remained low at 42% for JAKi and 24% for IL-6 inhibitors, respectively [24]. Furthermore, a prospective study of 71 patients with VEXAS syndrome evaluated disease trajectories with such therapies and reported no significant reduction in glucocorticoid exposure or risk of hematologic disease progression, identifying the continued challenges in the management of patients with VEXAS syndrome [25].

In the present survey, azacitidine was used most typically in the MDS setting. There is emerging use of azacitidine in VEXAS syndrome both with or without concurrent MDS [24, 26]. Obtaining cytokine-directed therapies and azacitidine remains a major issue with the need for special or and compassionate access schemes. Concerningly, for JAKi and azacitidine, patient payment was reported by 9% and 16.3% of the clinicians, respectively, indicating a potential source of financial toxicity to the patients [27]. There is also a need to develop treatment response criteria in VEXAS syndrome particularly given that a third of the clinicians reported trialing systematic treatments for 1–2 months before declaring inefficacy and switching to alternate agent.

Allo-HSCT remains the only cure for VEXAS syndrome, being able to eliminate the *UBA1* clone [28]. There is uncertainty on clear indications for allo-HSCT in VEXAS syndrome and many reports are from retrospective experience [8]. In this present survey, only a limited proportion of clinicians declared accessibility to transplant, further highlighting barriers to optimal management options.

There are several limitations of this survey to be considered. Firstly, there was a selection bias with high representation of expert clinicians in European centers as part of consolidated VEXAS networks (e.g., France, Italy, Spain). The opinions of these clinicians and the capabilities of the centers in which they practice may not be able to be extrapolated to other countries and institutions globally. Secondly, as some of the questions were optional, there were incomplete or missing data. Thirdly, the questions may have been elaborated to capture more insights; however, a pragmatic approach to the number and complexity of the questions was adopted to maximally encourage survey engagement and dissemination. Moving forward, a multinational collaboration in contributing to a worldwide registry would be helpful to enable cohorts of sufficient sample size to be analyzed, including adequately powered sub-group analysis according to age, ethnicity, phenotypic cluster, and specific *UBA1* variant.

## Conclusion

In summary, VEXAS syndrome is still an under-recognized condition managed with heterogeneous treatment paradigms around the world. This survey has provided a detailed summary of clinician practices, identifying crucial areas for which guidance is needed, spanning from laboratory workup to treatment allocation. Importantly, the absence of on-label drugs has revealed the potential burden of financial toxicity for patients, emphasizing an urgent need for clinical trials in VEXAS syndrome to identify effective, accessible, and targeted molecular therapies. A follow-up survey over the next 5 to 10 years, involving clinicians and patient partners of diverse backgrounds, will provide rich comparison data, and hopefully capture the advent of rigorous data-driven clinical guidelines.

## Supplementary Information

Below is the link to the electronic supplementary material.

ESM 1 (DOCX 48.0KB)

## Data Availability

Data is available within the main manuscript and supplemental material. Additional data will be made available upon reasonable request.
