# Supplementary material for: A cross-sectional survey on VEXAS syndrome: insights from a global expert panel
Source: Clin Rheumatol. 2025 Aug 9;44(10):4385–93. doi: 10.1007/s10067-025-07617-3 (PMC12518442; doi:10.1007/s10067-025-07617-3)
Supplement: Supplementary file 1 — (DOCX 48.0KB) [file 10067_2025_7617_MOESM1_ESM.docx]

**A cross-sectional survey on VEXAS syndrome: insights from a global expert panel**

**Supplementary Material**

*Supplementary Table:* Geographical location of clinicians who participated in survey.

| **Clinicians’ respondents: 74**  **Africa:** 1   - Morocco: 1 (1.4%)   **Asia:** 6   - China: 1 (1.4%) - India: 2 (2.7%) - Israel: 1 (1.4%) - Japan: 2 (2.7%)   **Australasia**: 6   - Australia: 6 (8.1%)   **Europe:** 51   - Belgium: 1 (1.4%) - Czech Republic: 1 (1.4%) - France: 8 (10.8%) - Germany: 2 (2.7%) - Italy: 19 (25.7%) - Norway: 2 (2.7%) - Poland: 2 (2.7%) - Portugal: 2 (2.7%) - Romania: 1 (1.4%) - Spain: 10 (13.5%) - The Netherlands: 1 (1.4%) - United Kingdom: 2 (2.7%)   **North America:** 9   - Canada: 2 (2.7%) - Mexico: 1 (1.4%) - United States of America: 6 (8.1%)   **South America:** 1   - Uruguay: 1 (1.4%) |
| --- |

*Supplementary File: Cinlician’s survey questions*

**Section 1: Introduction**

To better understand VEXAS disease and management around the world, the following survey is being proposed which evaluates: clinician background, molecular biology testing methods and local capabilities, and physician’s preferences on therapeutic choices.

The aim is to provide a screenshot of the global VEXAS cartography in terms of diagnostic capabilities and disease awareness.

Email address*: *free text*

^* All email addresses will be kept confidential.^

Consent: There are no identifiable factors for this survey, and before proceeding with the survey please indicate whether you consent to this survey: Y/N

**Section 2: Clinician background**

1. Please select your specialty*: Haematology, Rheumatology, Immunology, Internal Medicine, Other

2. Please indicate your country of practice*: *free text*

**Section 3: Clinical Cohort**

1. In 2024, how many VEXAS patient(s) did you manage*: 1-4, 5-9, 10-14, 15-29, >30, Other

2. On average, how frequently did you review your patients*: <4-weeks, 4-5weeks, 6-7weeks, 8-9weeks, >10weeks

3. Do you visit VEXAS patients in liaison with another medical specialist in a dedicated outpatient clinic?* Y/N

4. If you review VEXAS patients in liaison with another medical specialist, please indicate specialty/ specialties involved: *free text*

**Section 4: Molecular biology testing**

1. Is *UBA1* diagnostic testing performed in your institution?* Y/N

2. Which technique do you use for diagnosis of VEXAS?* Sanger sequencing of exon 3, Sanger Sequencing of exon 3 and other regions of *UBA1* gene, ddPCR of Met41Leu/Val/Thr, ddPCR of Met41Leu/Val/Thr and other hotspots, NGS of *UBA1* exon 3, NGS of the entire *UBA1* gene, clinical exome, other

3. If you answered other in the above question, please specify: *free text*

4. Do you perform *UBA1* on*: peripheral blood only, peripheral blood and bone marrow, bone marrow only

5. On average, what is your turn around time for *UBA1* diagnostic testing*: <2 weeks, 2-4 weeks, 6-12 weeks, >12 weeks

6. Is your centre able to offer *UBA1* Variant Allele Frequency (VAF) testing on patients?* Y/N;

7. If your centre is able to offer UBA1 VAF testing on patients, please specify with which technique? *free text*

8. If your centre is able to offer UBA1 VAF testing on patients, please specify frequency of testing*: once only i.e., at diagnosis, every 3 months, every 6 months, every 12 months, other

9. If you answered ‘other’ please specify: *free text*

10. If your centre is not able to offer *UBA1* VAF testing, are you able to send the sample for UBA1 VAF testing to a referral centre? Y/N/ NA

**Section 5: Thrombosis and ancillary testing**

1.Which anticoagulants do you use in patients with VEXAS developing thrombosis?* Direct oral anticoagulants (DOACs) only, Vitamin K antagonist only, both, other

2.How long will you consider to anticoagulated patients with VEXAS?* Indeterminate as they have VEXAS, according to the type of thrombosis (provoked/ unprovoked) and thrombophilia panels

3. Do you test for antiphospholipid antibodies (APLAb) in patients with VEXAS?* Y/ Not routinely

4. If found positive to APLAb on two separate occasions four months apart, do you stop DOACs and switch to vitamin K antagonists? Y/ N as they have VEXAS

**Section 6: Ancillary Testing**

1. Which laboratory investigations(s) do you routinely assess in patients with VEXAS at each visit (check all that apply)*: C-reactive protein, erythrocyte sedimentation rate, full blood count, glucose metabolism, hepatic function, renal function, serum electrophoresis

2. In your opinion, which ancillary laboratory investigation gives a better idea of VEXAS activity (not considering *UBA1* VAF)?* C-reactive protein, erythrocyte sedimentation rate, full blood count, other

3. If other, please specify: *free text*

4. Do you routinely perform imaging in patients with VEXAS?* Y/ N, only if clinically indicated

5. If you perform imaging, please specify: *free text*

**Section 7: Management**

*i.Corticosteroids*

1. Please indicate the initial daily dose prednisolone (or equivalent) you usually start a patient with VEXAS and active disease*: 2mg/kg/ 1.5mg/kg, 1mg/kg, 0.5mg/kg, other

2. Please indicate the maximum time used at highest dose of prednisolone/ equivalent*: <2 weeks, 2 weeks, 4 weeks, > 4 weeks

3. What is the percentage of your patients with VEXAS on long term treatment with prednisolone (i.e., >6 months)*: >50%, >80%, >90%, All of them have a residual prednisolone dose

4. What is the overall maximum long-term daily dose of prednisolone/ equivalent*: 5mg, 10mg, 15mg, 20mg, other

5. For acute flares of VEXAS, which route of administration is your practice?*: Intravenous, oral, both intravenous and oral

6. Regarding concurrent prophylaxis please select which applies*: antifungal, antibacterial and antiviral medications, antifungal and antibacterial, antifungal, antibacterial, antiviral, antiviral and antifungal, antiviral and antibacterial, other

7. If you selected ‘other’ for concurrent prophylaxis, please specify: *free text*

*ii.Systemic immune-modulatory treatment:*

1. Systemic immunomodulatory treatment is added only if*: no response to corticosteroid monotherapy, upfront

2. Please indicate the immunomodulatory treatment(s) in order of your preference or experience: *free text*

3. How long would a trial of systemic immunomodulatory drug before declaring drug ineffective and switching to an alternate agent: <1 month, 1-2 months, 3-6 months, >6 months

4. JAK-i: If selected, which is used, and usual starting dose: *free text*

5. JAK-i: if used, how is this medication obtained: special access scheme, compassionate access, company provision, patient payment, other

6. Azacitidine: Do you use it in patients with concomitant MDS only*? Y/N

7. Azacitidine: If not used in MDS, how is this medication obtained: special access scheme, compassionate access, company provision, patient payment, other

iii.Transplant:

1. Does your institution have access to a hematopoietic stem cell transplant service? Y/N

2. Has your department successfully completed a transplant for VEXAS: Y/N/ NA

3. If your department has successfully completed a transplant for VEXAS, did you consider transplant also in patients with VEXAS alone without MDS?: Y/N/ NA

**Section 8: Thank you for your participation in this survey. We are grateful for your time in completing this survey.**

Please feel free to leave any comments: *free text*

*Mandatory question
